# Microwave solvothermal synthesis and characterization of manganese-doped ZnO nanoparticles

**DOI:** 10.3762/bjnano.7.64

**Published:** 2016-05-19

**Authors:** Jacek Wojnarowicz, Roman Mukhovskyi, Elzbieta Pietrzykowska, Sylwia Kusnieruk, Jan Mizeracki, Witold Lojkowski

**Affiliations:** 1 Institute of High Pressure Physics, Polish Academy of Sciences, Sokolowska 29/37, 01-142 Warsaw, Poland; 2 Faculty of Materials Science and Engineering, Warsaw University of Technology, Woloska 141, 02-507 Warsaw, Poland

**Keywords:** manganese-doped zinc oxide nanoparticles, microwave solvothermal synthesis (MSS), characterization techniques of nanomaterials, physical properties of Mn^2+^-doped ZnO nanoparticles

## Abstract

Mn-doped zinc oxide nanoparticles were prepared by using the microwave solvothermal synthesis (MSS) technique. The nanoparticles were produced from a solution of zinc acetate dihydrate and manganese(II) acetate tetrahydrate using ethylene glycol as solvent. The content of Mn^2+^ in Zn_1−_*_x_*Mn*_x_*O ranged from 1 to 25 mol %. The following properties of the nanostructures were investigated: skeleton density, specific surface area (SSA), phase purity (XRD), lattice parameters, dopant content, average particle size, crystallite size distribution, morphology. The average particle size of Zn_1−_*_x_*Mn*_x_*O was determined using Scherrer’s formula, the Nanopowder XRD Processor Demo web application and by converting the specific surface area results. X-ray diffraction of synthesized samples shows a single-phase wurtzite crystal structure of ZnO without any indication of additional phases. Spherical Zn_1−_*_x_*Mn*_x_*O particles were obtained with monocrystalline structure and average particle sizes from 17 to 30 nm depending on the content of dopant. SEM images showed an impact of the dopant concentration on the morphology of the nanoparticles.

## Introduction

Nanotechnology has triggered a new global industrial revolution of the 21st century [1]. At present it is the leading technology in various research fields such as chemistry, physics, biology, medicine, materials and biomedical engineering, optoelectronics and interdisciplinary fields. It is a technology that enables testing, controlling, producing and using structures at least one dimension of which is below 100 nanometres [2]. It enables the use of nanomaterials for creating innovative products, devices and complex systems that employ the properties of materials on the nanoscale [3]. Current research of the applications of nanotechnology in optoelectronics focuses on the control of the physical properties of metal oxides semiconductors.

Zinc oxide (ZnO) is a II–VI semiconductor characterised by a wide band gap of 3.3 eV and a high exciton binding energy of circa 60 meV [4]. ZnO is used in optoelectronic devices, solar cells, data carriers, light emitting diodes (LEDs), gas sensors, thermoelectric devices, varistors, TFT display windows and laser technology [5–7]. ZnO displays pyroelectric and piezoelectric properties, thanks to which it is used in electroacoustic devices [8]. It is a biocompatible material used for producing biosensors and in drug delivery applications [9]. Thanks to antibacterial activity, matting and hiding properties, as well as bleaching properties, it is applied in the pharmaceutical and cosmetic industry to produce creams, dressings, powders, baby powders and toothpastes. In paediatric dentistry, it is the primary ingredient of the temporary filling material [10]. It is also a popular mineral filter against UVA and UVB radiation [11–13].

**Figure 1:**
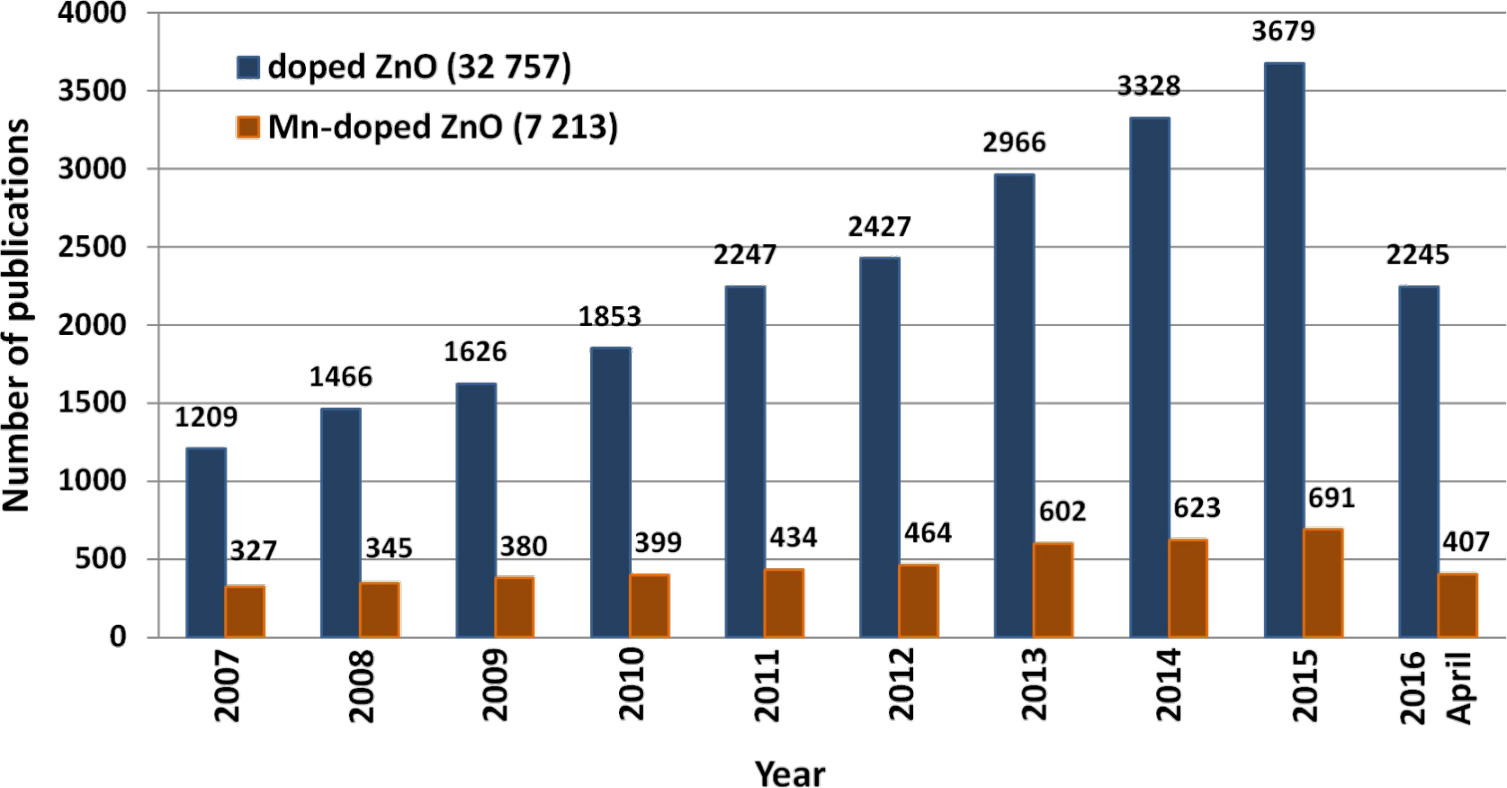
The number of scientific publications referring to the search of “doped ZnO” and “Mn doped ZnO” phrases published in the period 2007–2015. Source: ScienceDirect (accessed April 27, 2016).

The search for “doped ZnO” and “Mn doped ZnO” phrases in the “ScienceDirect” scientific search engine yielded 32,757 and 7,213 matches, respectively, (April 2016) in various research fields. The number of published scientific papers confirms the growing interest in Mn^2+^-doped zinc oxide (Figure 1). It is generally known that selective doping enables controlling the semiconductor properties of ZnO, such as forbidden band or conductivity. The diversity of ZnO modifications by doping with various transition metal ions (e.g., Co, Mn, Ni, Fe, Cr, V) considerably increases the capabilities of applying that material in electronics, spintronics and optoelectronics [14–16].

**Figure 2:**
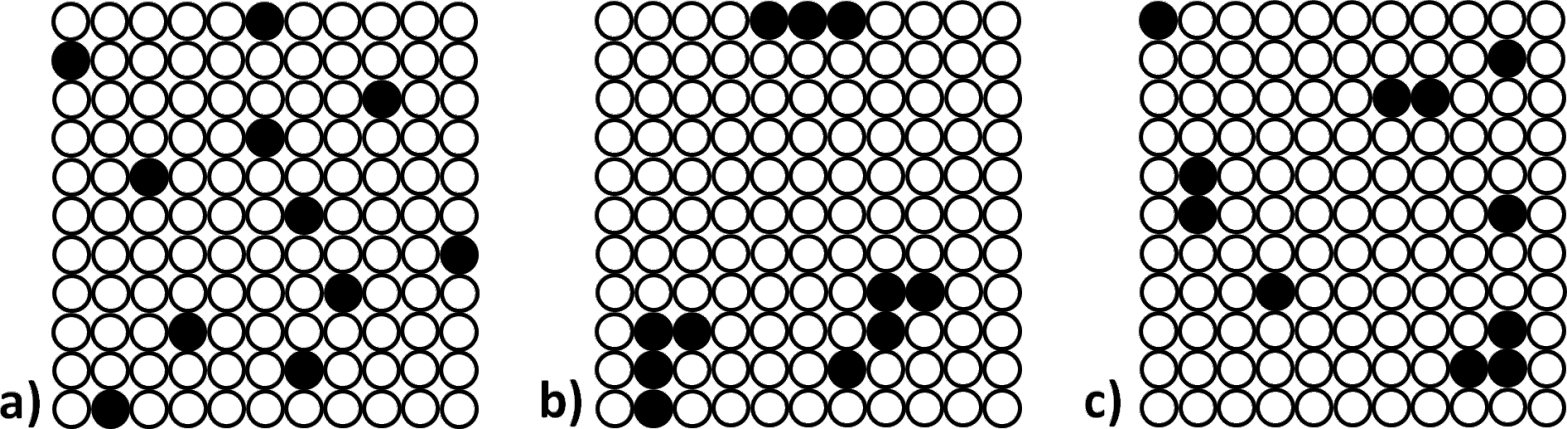
Indicative examples of the possible distribution of Mn^2+^ dopant in zinc oxide crystalline lattice. Black circles: Mn^2+^ dopant, white circles: ZnO. a) Unclustered distribution of dopant; b) clustered distribution of dopant; c) random distribution of dopant.

Papers related to the magnetic properties of Mn^2+^-doped ZnO focus on the impact of homogeneity, phase purity and dopant content on the properties [17–18]. In the case of substituting Zn^2+^ atoms by Mn^2+^ atoms in a ZnO crystalline lattice, an increase in the value of lattice parameters was observed [19–20]. The ionic radius of Mn^2+^ is 0.82 Å [21], which is greater than that of Zn^2+^ being 0.74 Å [18]. Therefore, Zn^2+^ can be substituted to a limited extent by Mn^2+^ [22]. Numerous scholars have described obtaining Mn^2+^-doped zinc oxide, which displays ferromagnetic properties at room temperature, in their papers [23–26]. However, there are many contradicting or even controversial publications on this topic [27–30]. Depending on the method of obtaining, Zn_1−_*_x_*Mn*_x_*O may display either ferromagnetic [31] or paramagnetic properties [32]. Various pieces of literature reporting magnetic properties of Zn_1−_*_x_*Mn*_x_*O NPs result from the impact of the synthesis method on: the heterogeneous distribution of Mn^2+^ ions in the ZnO crystalline lattice (e.g., formation of clusters [31]), the precipitation of foreign phases [33–34], the stoichiometry; the presence of oxygen vacancies [35–36], and defects of the crystalline lattice. When the synthesis or calcination of Zn_1−_*_x_*Mn*_x_*O was carried out in an oxidising environment and the absence of foreign phases was observed, the obtained material exhibited paramagnetic properties [29,33,37]. Whereas, when Zn_1−_*_x_*Mn*_x_*O was synthesised or calcinated for too long or at an overly high temperature, in an oxidising atmosphere, foreign phases in the form of MnO, MnO_2,_ Mn_2_O, Mn_3_O_4_, as well as products of the reaction of ZnO with Mn*_x_*O*_y_* such as ZnMnO_3_ and ZnMn_2_O_4_ were precipitated in the material. The obtained Zn_1−_*_x_*Mn*_x_*O materials were characterised by either ferromagnetic or paramagnetic properties depending on the presence of foreign phases [33,36,38–39]. This results from different magnetic properties of Mn*_x_*O*_y_* and metallic Mn. Metallic manganese is antiferromagnetic, while many alloys of manganese, in which the average Mn–Mn distance is greater than that of metallic manganese, are ferromagnetic [40]. MnO, Mn_2_O_3_ and MnO_2_ are antiferromagnetic [41], while Mn_3_O_4_ is ferromagnetic [42–43]. ZnMnO_3_ is paramagnetic [44–45]. But when a reducing environment was selected for sample calcination, also precipitations of ZnMnO_3_ and ZnMn_2_O_4_ phase emerged in Zn_1−_*_x_*Mn*_x_*O and the material displayed only ferromagnetic properties [36]. Ferromagnetic properties of Zn_1−_*_x_*Mn*_x_*O are explained by the presence of oxygen vacancies, manganese in various oxidation states (II–IV), and dopant clusters. The formation of clusters of Mn^2+^ dopant in ZnO NPs, e.g., on the particle surface, may lead to a various kind of magnetic coupling of atoms with their adjacent atoms [46]. This, in turn, leads to different magnetic properties than those encountered in nanomaterials with the same dopant content but with a different distribution of ions or dopant clusters in the crystalline lattice (Figure 2) [47].

Despite the efforts made by numerous research groups and despite the development of new synthesis methods, obtaining nanomaterials with reproducible properties proves a very complex and problematic task. Obtaining Zn_1−_*_x_*Mn*_x_*O nanomaterials with reproducible optoelectronic and magnetic properties remains an unsolved issue. Differing properties of the obtained Zn_1−_*_x_*Mn*_x_*O result from the complexity of chemical reactions and the limitations of the currently employed methods. The lack of simultaneous control over chemical composition, stoichiometry, dopant homogeneity, particle size distribution, shape, phase purity, surface modification and agglomeration, makes it difficult to obtain NPs [22]. The primary cause of the lack of reproducibility of magnetic properties in the case of Zn_1−_*_x_*Mn*_x_*O is the lack of control over the doping impact on the formation of oxygen vacancies, crystalline lattice defects and dopant clusters [48]. It is presumed that there are several competitive chemical reactions in the reaction of Zn_1−_*_x_*Mn*_x_*O synthesis, such as the oxidisation of Mn^2+^ ions, the formation of Mn*_x_*O*_y_*, the reduction of Mn^2+^ ions to metallic Mn, and the reaction of Mn*_x_*O*_y_* with ZnO leading to the formation of spinels (ZnMnO_3_, ZnMn_2_O_4_). These reactions may occur simultaneously depending on the synthesis method, the synthesis parameters and the method of precursor preparation. This explains the various reports about obtaining Zn_1−_*_x_*Mn*_x_*O with different and in parts contradictory properties. The relevant literature describes several methods for the synthesis of Zn_1−_*_x_*Mn*_x_*O nanomaterials, in particular coprecipitation [49], coprecipitation–calcination [29,50–51], combustion [52], sol–gel [7,31,33,53–54], spin coating [19–20], pulsed laser deposition [26], reactions in the solid state [35,55], thermal evaporation [56], and thermal evaporation vapour-phase deposition [30]. However, two of the most promising methods, which are still being developed are hydrothermal and solvothermal synthesis [16,57]. The growing popularity of these methods is proved by the emergence of new types of reactors, e.g., microwave stop-flow and continuous-flow in supercritical water [57–63]. Microwave solvothermal synthesis (MSS) is quicker, purer, and more energy and cost efficient than conventional synthesis methods. The microwave radiation employed is a highly effective method of providing energy to the reaction chamber, which results in a more uniform and rapid heating in comparison with traditional methods of heat transfer [64]. The MSS method is a “wet chemistry” method. This means that the precursor is obtained from solutions of substrates, which are mixed in precisely determined quantities, and the synthesis is carried out under controlled conditions (temperature, pressure, time). The synthesis product is a precipitate, which is mainly subjected to filtering, rinsing and drying [65]. The advantages of the microwave solvothermal synthesis are purity (Teflon reaction chamber, contactless heating method), short process duration, precise control over parameters (time, pressure), product homogeneity (volumetric heating), control over dopant content, high efficiency, and surface modification [47]. MSS products are characterised by homogeneous morphology, purity, narrow size distribution and low agglomeration.

The present paper contains an attempt to obtain ZnO nanoparticles with a Mn^2+^ dopant content of up to 25 mol %. For the synthesis we selected an organic solvent with weak reducing properties to prevent the precipitation of foreign phases, which is caused above all by the changed oxidisation state of Mn^2+^ ions. The microwave solvothermal synthesis was selected for obtaining Zn_1−_*_x_*Mn*_x_*O. It was shown before by us that the MSS method led to fully crystalized Co^2+^-doped ZnO without foreign phases and with a uniform particle size and shape [66].

## Experimental

### Substrates

Zinc acetate dihydrate (Zn(CH_3_COO)_2_·2H_2_O), analytically pure*,* SKU: 112654906-1KG; ethylene glycol (1,2-ethanediol, C_2_H_4_(OH)_2_), pure, SKU: 114466303-5L and manganese(II) acetate tetrahydrate (Mn(CH_3_COO)_2_·4H_2_O), analytically pure, SKU: 116167509-100g were purchased from Chempur. The reagents were used without additional purification.

#### Synthesis of Zn_1−_*_x_*Mn*_x_*O NPs

Zn_1−_*_x_*Mn*_x_*O NPs were obtained by the MSS method. The precursor was obtained by dissolving powder mixtures, composed of 1, 5, 10, 15, 20, 25 mol % of manganese acetate in zinc acetate, in ethylene glycol (Table 1). The reference sample of ZnO was obtained from the precursor without adding the Mn^2+^ doping. The mixture of the acetates in glycol (75 mL) was heated to 70 °C and stirred using a magnetic stirrer (SLR, SI Analytics, Germany) until the components were completely dissolved. After cooling down to ambient temperature the solution was poured to the Teflon reaction chamber with a total volume of 110 mL [66].

**Table 1:** Composition of precursors of Zn_1−_*_x_*Mn*_x_*O synthesis.

name of precursor	*c*(Zn(CH_3_COO)_2_·2H_2_O) [mol/L]	*c*(Mn(CH_3_COO)_2_∙4H_2_O) [mol/L]	solvent

ZnO	0.3254	0	ethylene glycol
Zn_0.99_Mn_0.01_O	0.3254	0.0033	
Zn_0.95_Mn_0.05_O	0.3254	0.0171	
Zn_0.90_Mn_0.10_O	0.3254	0.0362	
Zn_0.85_Mn_0.15_O	0.3254	0.0574	
Zn_0.80_Mn_0.20_O	0.3254	0.0814	
Zn_0.75_Mn_0.25_O	0.3254	0.1085	

The synthesis reaction initiated by microwave radiation was carried out in a microwave reactor (Model 02-02, 600 W, 2.45 GHz, ERTEC, Poland) at a temperature of 200 °C. The synthesis of Mn^2+^-doped zinc oxide in ethylene glycol can be described through the following equation:


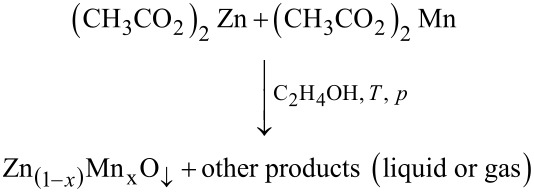


The reaction duration was 25 min and afterwards the reactor chamber was cooled down for 20 min to reach a temperature below 100 °C in order to safely remove the reaction vessel. After the synthesis the suspension was subjected to sedimentation and decantation. The sediment was rinsed three times with deionised water (HLP 20UV, Hydrolab, Poland). In order to obtain a dry Zn_1−_*_x_*Mn*_x_*O powder, the sediment was suspended in water and rapidly cooled down with liquid nitrogen and subsequently dried in a freeze dryer (Lyovac GT-2, SRK Systemtechnik GmbH, Germany).

### Characterisation methods

#### X-ray powder diffraction

Diffraction patterns of the X-ray powder diffraction (XRD) were gathered at room temperature in the 2 theta range from 10 to 100° with a step width of 0.02° (Cu Kα1, X’Pert PRO, Panalytical, Netherlands). The crystalline lattice parameters were determined by the Rietveld method. Diffraction patterns were fitted in Fityk software, version 0.9.8, using the implemented Pearson 7 feature. Based on the diffraction patterns, the size of crystallites was determined (*D_hkl_*) using Scherrer’s formula (Equation 1), where *D_hkl_* is the volume weighted crystallite size [nm]; β is the FWHM of the *hkl* diffraction peak [rad]; *K* is a constant shape factor (*K* ≈ 1), λ is the X-ray wave length [nm] and θ*_hkl_* is the Bragg diffraction angle.

[1]
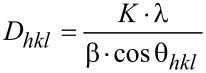


#### Crystallite size distribution

The analysis of XRD peak profiles was performed using the analytical formula for polydispersive powders [67]. While the Scherrer method provides a single size parameter, this technique provides four parameters: average crystallite size, error of the average crystallite size, dispersion of size and error of dispersion of sizes. Hence, a full crystallite size distribution curve with its errors is obtained. The on-line tool Nanopowder XRD Processor Demo (http://science24.com/xrd/) is a webpage where diffraction files can be uploaded. Files are processed on a server to extract the crystallite size distribution for XRD peaks. Unlike the standard fitting, the tool does not act in the reciprocal space at all, but solves sets of equations in a few auxiliary spaces simultaneously. This allows for an analysis of XRD data with heavily convoluted reciprocal space peaks [68–69].

#### Measurement of density and specific surface area

Density measurements were carried out using a helium pycnometer (AccuPyc II 1340 FoamPyc V1.06, Micromeritics, USA), in accordance with ISO 12154:2014 at the temperature of 25 ± 2 °C. The density of a material obtained using helium pycnometry is called, e.g., skeleton density, pycnometric density, true density and helium density in the relevant literature. The specific surface area of NPs was determined using the analysis of nitrogen adsorption isotherm by the BET (Brunauer–Emmett–Teller) method (Gemini 2360, V 2.01, Micromeritics), in accordance with ISO 9277:2010. Prior to performing measurements of both density and specific surface area, the samples were subject to 2 h desorption in a desorption station (FlowPrep, 060 Micromeritics), at a temperature of 150 °C with a flow of helium of 99.999% purity. Based on the determined specific surface area and density, the average size of particles was determined, with the assumption that all particles are spherical and identical [70]. Equation 2 was used for calculating the average particle size, where *D* is the average diameter of the of particles [µm], *N* is a shape coefficient being (*N* = 6) for sphere, *SSA* is the specific surface area [m^2^/g] and ρ the density [g/cm^3^].

[2]
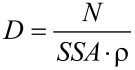


#### Morphologic characteristics

The morphology of NPs was determined using scanning electron microscopy (SEM) (ZEISS, Ultra Plus, Germany). The samples were coated with a thin carbon layer using a sputter coater (SCD 005/CEA 035, BAL-TEC, Switzerland).

#### Chemical composition analysis

The chemical composition analysis was carried out by using visible-light spectrophotometry (DR 3900, Hach Lange, Germany). The analysis of the content of Zn^2+^ ions was carried out by using the Zincon method No. 8009 (Hach Lange). The analysis of the content of Mn^2+^ ions was carried out by using the 1-(2-pyridylazo)-2-naphthol (PAN) method No. 8149 (Hach Lange). The chemical composition analysis was carried out by using inductively coupled argon plasma optical emission spectrometry (ICP-OES) (Thermo Scientific, iCAP model 6000, Great Britain).

Samples for the quantitative analysis were prepared as follows: 5 mg of powder was weighed in a 110 mL Teflon vessel and 15 mL of deionised water (HLP 20UV, Hydrolab, Poland) was added; then 6 mL of HNO_3_ was added and the solution subjected to one microwave heating cycle in a Magnum II reactor (600 W, 2.45 GHz, ERTEC, Poland). After cooling the sample volume was filled up to 50 mL with deionized water [66]. The pH of samples before the colorimetric analysis was adjusted in accordance with the recommendations of procedures No. 8009 and No. 8149.

Quantitative microanalysis was carried out through energy-dispersive X-ray spectroscopy (EDS) by using an EDS analyser (Quantax 400, Bruker, USA). Samples for EDS tests were pressed to pellets with a diameter of 5 mm.

## Results and Discussion

### Morphology

**Figure 3:**
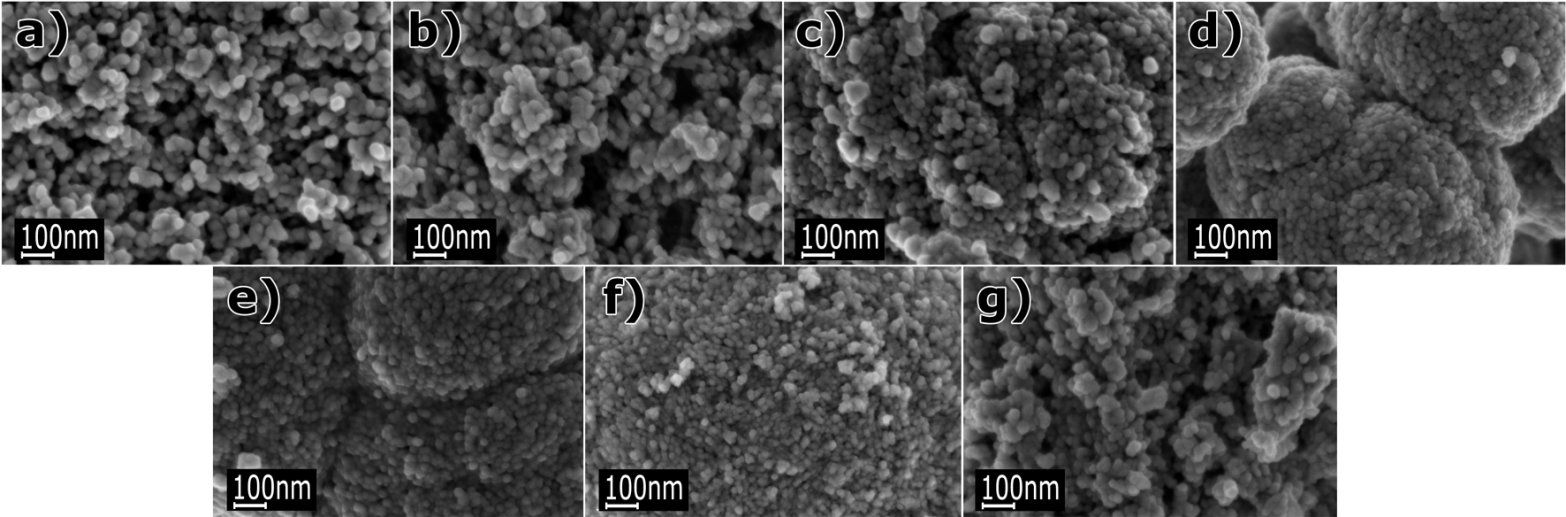
SEM images of NPs of Zn_1−_*_x_*Mn*_x_*O with dopant concentrations of: a) 0 mol %, b) 1 mol %, c) 5 mol %, d) 10 mol %, e) 15 mol %, f) 20 mol % and g) 25 mol %.

**Figure 4:**
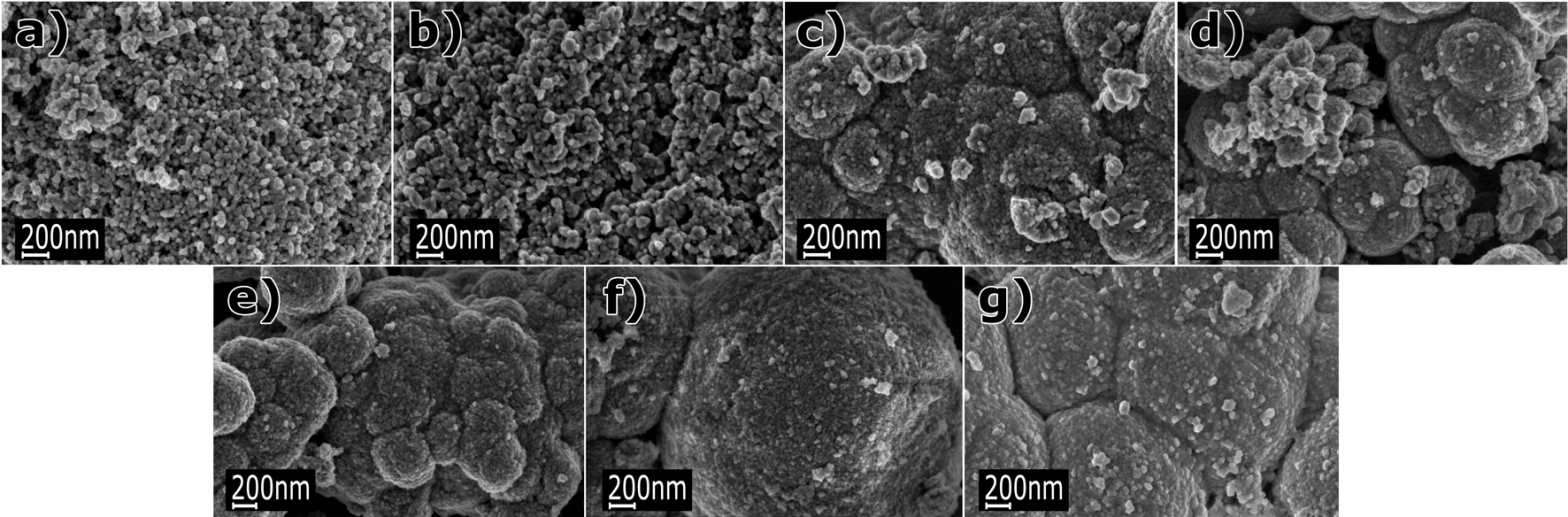
SEM images of agglomerates and conglomerates of Zn_1−_*_x_*Mn*_x_*O NPs with dopant concentrations of: a) 0 mol %, b) 1 mol %, c) 5 mol %, d) 10 mol %, e) 15 mol %, f) 20 mol % and g) 25 mol %.

Figure 3 and Figure 4 present representative SEM images of Zn_1−_*_x_*Mn*_x_*O NPs. They show particles with homogeneously distributed spherical shapes. An impact of the content of Mn^2+^ dopant on the morphology of Zn_1−_*_x_*Mn*_x_*O powder was observed. Powders with dopant contents of 5, 10, 15, 20 and 25 mol % are composed of dense structures forming conglomerates whose shape resembles the structure of a cauliflower (Figure 4). In the case of dopant contents of 0 or 1 mol %, loose nanopowders were obtained. It can be noticed that the average particle size decreases with increasing content of Mn^2+^ dopant in the NPs. The average particle size decreases from circa 30–35 nm to 15–25 nm with an increase in the content of Mn^2+^ dopant from 0 to 20 mol %. In the case of the powder with a dopant content of 25 mol %, the average particle size was circa 20–25nm. SEM tests reveal that each precursor composition must be treated individually. That means that synthesis parameters must be optimised for each precursor composition in order to eliminate the process of agglomeration and formation of conglomerates of Zn_1−_*_x_*Mn*_x_*O NPs.

### Phase composition

**Figure 5:**
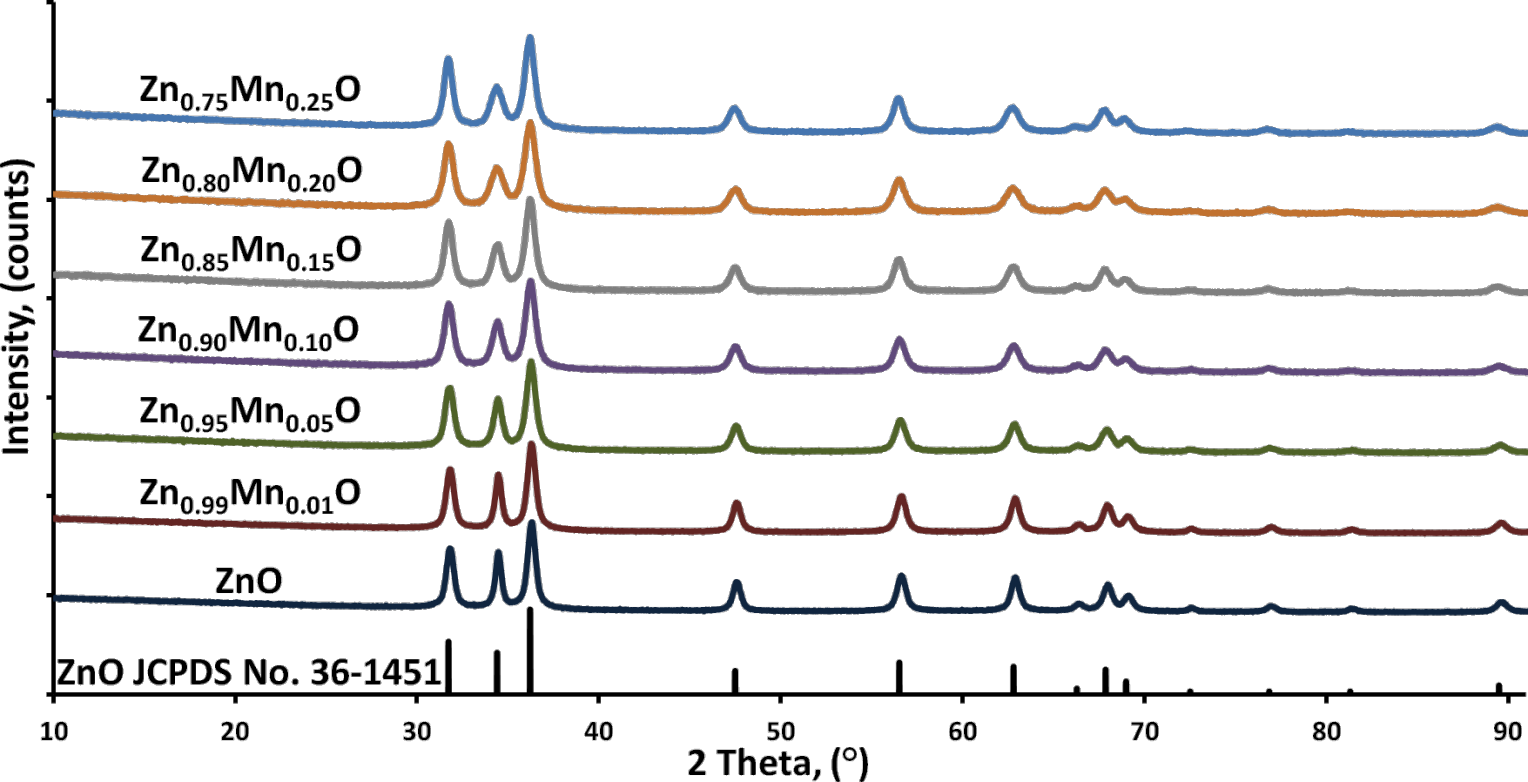
XRD diffraction patterns of Zn_1−_*_x_*Mn*_x_*O NPs, with the nominal dopant content in the solution being 0, 1, 5, 10, 15, 20, 25 mol % and its comparison with the standard pattern of wurtzite-phase ZnO (JCPDS No. 36-1451).

**Figure 6:**
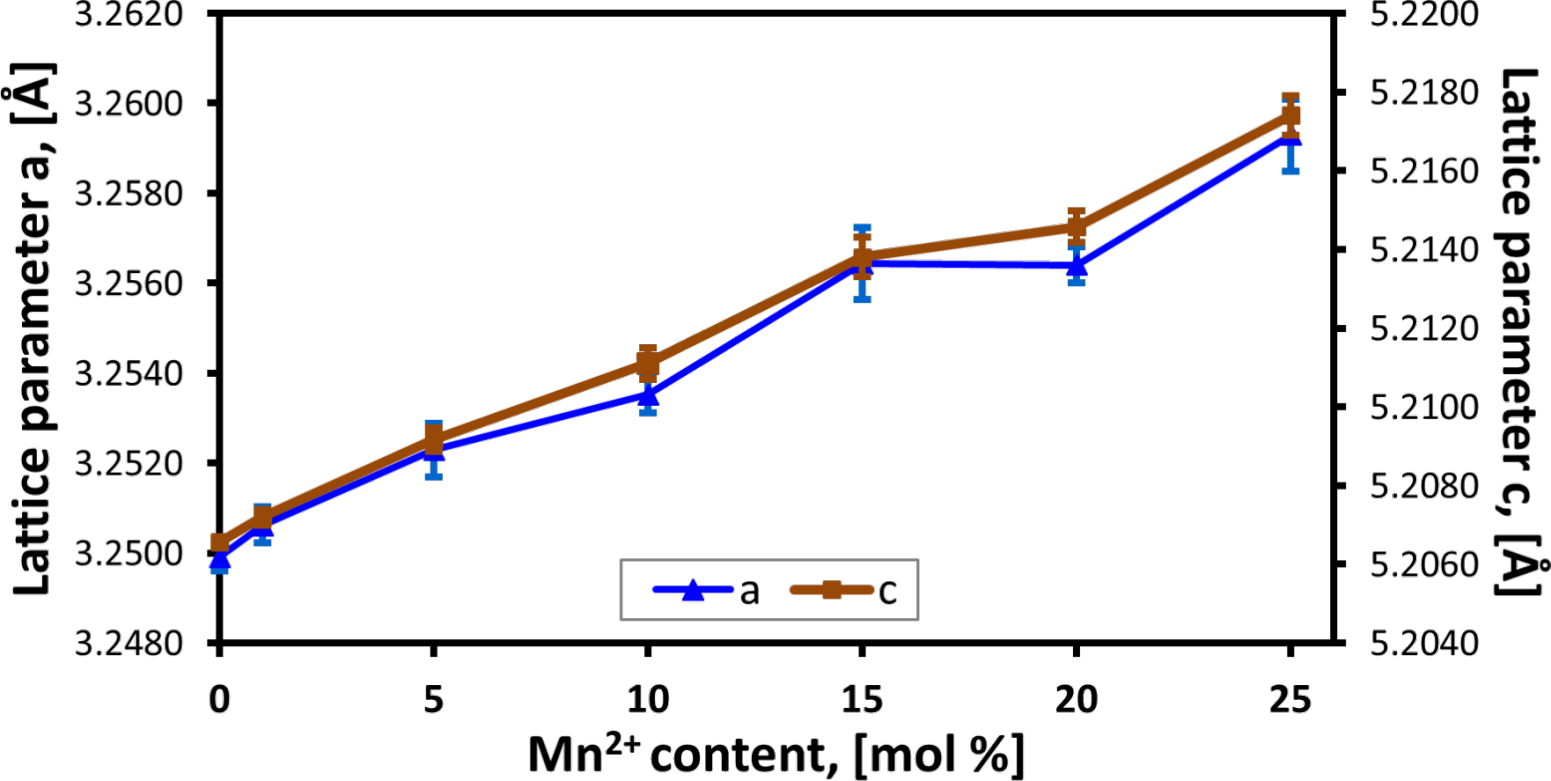
Lattice parameters versus nominal Mn^2+^ content of Zn_1−_*_x_*Mn*_x_*O samples.

**Figure 7:**
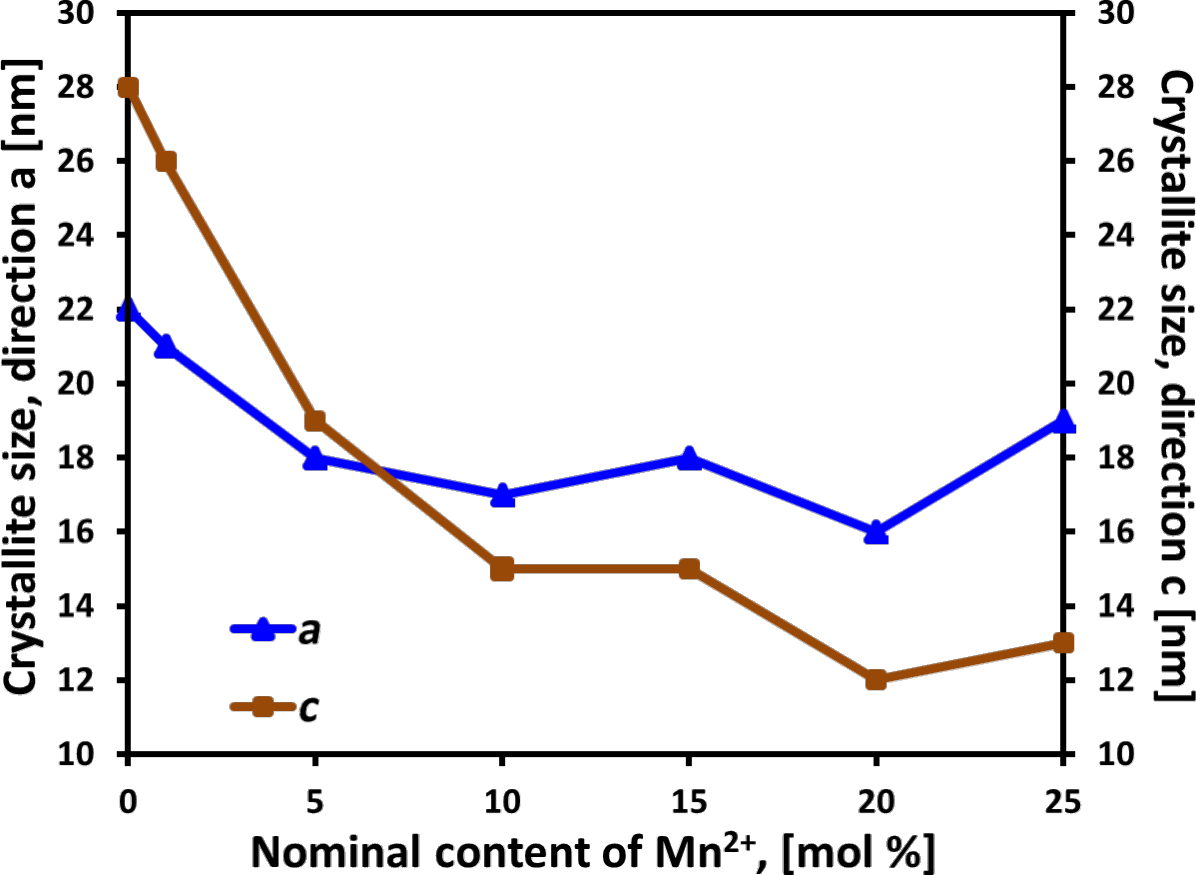
Dependence of the changes in crystallite size on the nominal content of Mn^2+^-dopant in Zn_1−_*_x_*Mn*_x_*O.

The XRD tests of the samples did not reveal a presence of foreign phases in the obtained Zn_1−_*_x_*Mn*_x_*O NPs, and all diffraction peaks can be attributed to the hexagonal phase ZnO (Figure 5). Zinc oxide is characterised by hexagonal wurtzite structure (JCPDS No. 36-1451, space group: *P*6_3_*mc*) with two lattice parameters *a* and *c* [4]. The parameters of ZnO crystalline lattice assume the values *a* = 3.2498 Å and *c* = 5.2066 Å, where its *c*/*a* ratio of 1.6021 is close to that of a close-packed hexagonal structure (hcp) *c*/*a* = 1.6330. MnO has rock salt structure (space group: *Fm−*3*m*) with the lattice parameter *a* = 4.4475 Å [71]. The radius of Mn^2+^ ions is bigger than that of Zn^2+^ ions by 0.08 Å, which explains the considerable changes in the lattice parameters (Table 2). Both lattice parameters *a* and *c* increase with increasing dopant content from 1 to 25 mol % (Figure 6). The results of the *c/a* lattice parameter ratio reveals that the change of the dopant content in Zn_1−_*_x_*Mn*_x_*O leads to a change in the proportions of the unit cell dimensions (Table 2). The obtained results of crystallite sizes *d_a_* and *d_c_* (Table 3, Figure 7) indicate that the increase of Mn^2+^-dopant content in ZnO leads to changes in proportions (asymmetry) of crystallite sizes. In line with the increase in Mn^2+^-dopant content, the size of the crystallites in the *c*-direction decreases (Figure 7). The *d_c_*/*d_a_* ratio can be interpreted as a change in the shape of Zn_1−_*_x_*Mn*_x_*O particles only if they are monocrystalline, which is the case for our samples (Table 3). If the *d_c_*/*d_a_* ratio assumes the value of 1, the shape of the Zn_1−_*_x_*Mn*_x_*O particles is spherical. If the *d_c_*/*d_a_* ratio assumes a value of different from 1, means particles exhibit a spherical/elliptical shape. Despite the presence of as much as 25 mol % dopant in the precursor solution, no foreign phases in the form of ZnMnO_3_, ZnMn_2_O_4_, metallic Mn, or Mn*_x_*O*_y_* were observed in the synthesis product, which proves that the organic solvent selected by us (ethylene glycol) prevented competitive reactions and a change of the oxidisation state of Mn^2+^ in the course of the synthesis. Nevertheless, it should be remembered that the limit of foreign phase detectability in the diffraction method (XRD) can be as high as 5–6 atom %.

**Table 2:** Lattice parameters and ratio of lattice parameters of the obtained Zn_1−_*_x_*Mn*_x_*O NPs.

sample	lattice parameters		lattice parameter ratio *c*/*a*	lattice parameter ratio *c*/*a* in hcp ZnO
	*a* ± σ [Å]	*c* ± σ [Å]		

ZnO (JCPDS No. 36-1451)	3.2498	5.2066	1.6021	1.6330
ZnO	3.2499 ± 0.0001	5.2066 ± 0.0003	1.6021	
Zn_0.99_Mn_0.01_O	3.2506 ± 0.0002	5.2072 ± 0.0004	1.6019	
Zn_0.95_Mn_0.05_O	3.2523 ± 0.0003	5.2092 ± 0.0006	1.6017	
Zn_0.90_Mn_0.10_O	3.2535 ± 0.0004	5.2111 ± 0.0004	1.6017	
Zn_0.85_Mn_0.15_O	3.2564 ± 0.0005	5.2138 ± 0.0008	1.6011	
Zn_0.80_Mn_0.20_O	3.2564 ± 0.0004	5.2146 ± 0.0004	1.6013	
Zn_0.75_Mn_0.25_O	3.2593 ± 0.0005	5.2174 ± 0.0008	1.6008	

**Table 3:** Crystallite sizes and size ratios of the obtained Zn_1−_*_x_*Mn*_x_*O NPs.

sample	average crystallite size, calculated through Scherrer’s formula		size ratio *d_c_*/*d_a_*
	*d_a_* [nm]	*d_c_* [nm]	

ZnO	22	28	1.273
Zn_0.99_Mn_0.01_O	21	26	1.238
Zn_0.95_Mn_0.05_O	18	19	1.056
Zn_0.90_Mn_0.10_O	17	15	0.882
Zn_0.85_Mn_0.15_O	18	15	0.833
Zn_0.80_Mn_0.20_O	16	12	0.750
Zn_0.75_Mn_0.25_O	19	13	0.684

### Chemical composition and impact of dopant on the colour of NPs

**Figure 8:**
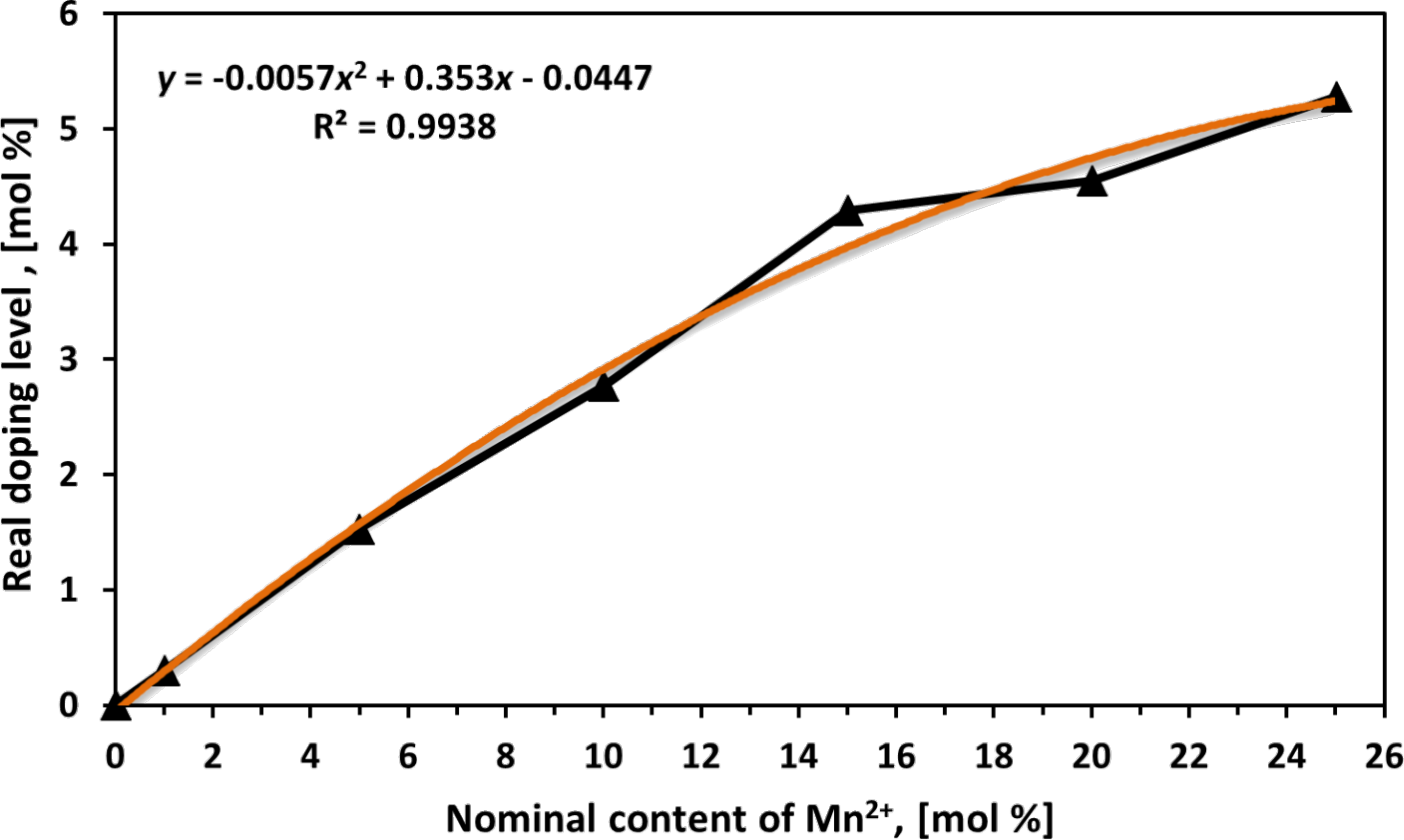
Correlation between the nominal content and the real content of Mn^2+^ dopant in ZnO. The results were obtained based on the ICP-OES analysis.

The real content (RC) of manganese dopant in Zn_1−_*_x_*Mn*_x_*O is presented in Table 4. The RC of dopant in Zn_1−_*_x_*Mn*_x_*O is smaller by circa 78% than the expected value of dopant arising from the nominal content (NC) of the dopant in the prepared precursor solution. The difference in sizes of Zn^2+^ and Mn^2+^ ionic radii is the reason for the low doping efficiency of Zn_1−_*_x_*Mn*_x_*O NPs. The chemical composition analysis was carried out by three methods: ICP-OES, colorimetry and X-ray microanalysis. The most often used methods in the relevant literature are ICP-OES and X-ray microanalysis. All the employed methods provided mutually different results (Table 4). This resulted from the limitations and accuracy of the various analytical techniques in relation to the quantitative determination of zinc and manganese. ICP-OES analysis is regarded as the most accurate and precise method. Figure 8 describes the correlation between NC in the precursor and RC of Mn^2+^ dopant in NPs. The average efficiency of doping during the synthesis of Zn_1−_*_x_*Mn*_x_*O in ethylene glycol was merely 22 mol %, being four times smaller than in the case of doping ZnO with Co^2+^ ions [66]. The derived formula (*y* = −0.0057*x*^2^ + 0.353*x* − 0.0447, where *y* is the real and *x* is the nominal dopant content) enables the control of RC of the dopant in Zn_1−_*_x_*Mn*_x_*O NPs at the stage of precursor preparation. The nominal contents of manganese dopant in Zn_1−_*_x_*Mn*_x_*O were used for denominating the samples in this paper.

**Table 4:** Analysis of chemical composition of Zn_1−_*_x_*Mn*_x_*O samples.

name of sample	actual content of dopant, mol %					
	colorimetric analysis [mol %]		EDS		ICP-OES	
	zinc	manganese	zinc	manganese	zinc	manganese

Zn_0.99_Mn_0.01_O	99.41	0.59	99.86	0.14	99.70	0.30
Zn_0.95_Mn_0.05_O	98.63	1.37	98.73	1.27	98.47	1.53
Zn_0.90_Mn_0.10_O	97.74	2.26	97.58	2.42	97.23	2.77
Zn_0.85_Mn_0.15_O	97.04	2.96	96.06	3.78	95.45	4.29
Zn_0.80_Mn_0.20_O	96.53	3.47	96.22	3.94	95.71	4.55
Zn_0.75_Mn_0.25_O	96.03	3.97	93.89	6.11	94.72	5.28

**Figure 9:**
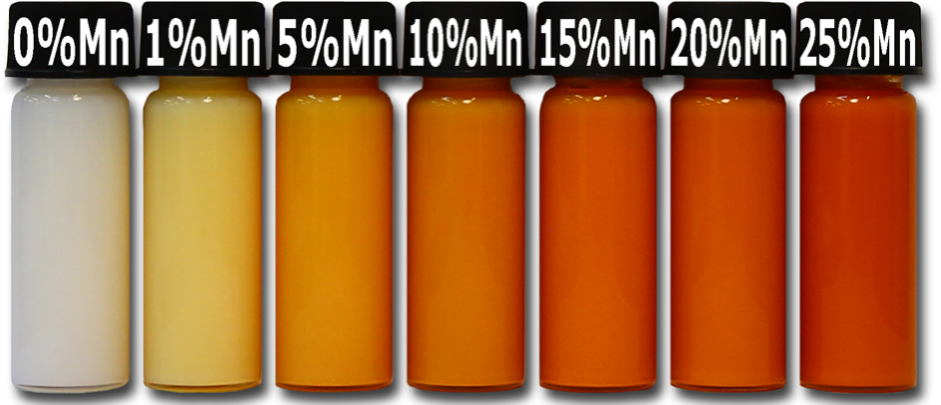
Visual comparison of changes in colours of suspensions of Zn_1−_*_x_*Mn*_x_*O NPs depending on the dopant content.

**Figure 10:**
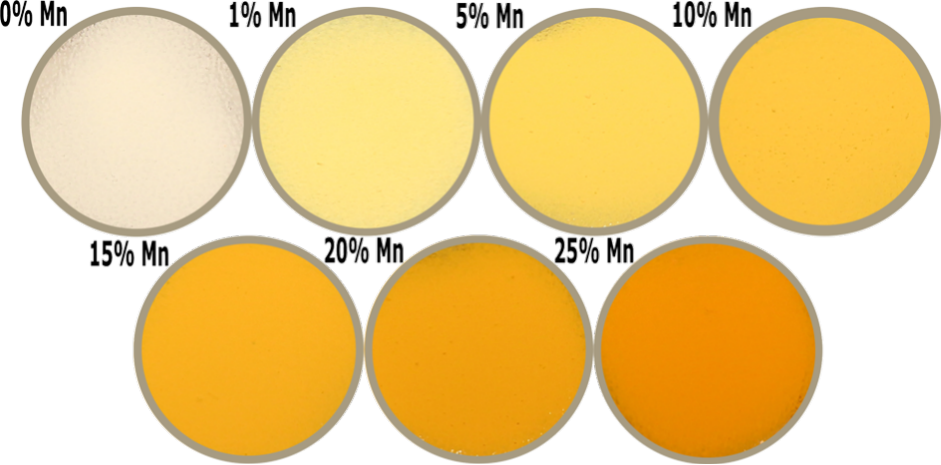
Visual comparison of changes in colours of dry powder of Zn_1−_*_x_*Mn*_x_*O NPs depending on the dopant content.

The doping of zinc oxide with Mn^2+^ ions leads to a change in the optical properties of the material, which is proved by the change of the sample colour (Figure 9 and Figure 10). The obtained Zn_1−_*_x_*Mn*_x_*O NPs, depending on the Mn^2+^-dopant content, have differing shades of yellow and orange. The intensity of NPs colour depends on the dopant content, which is illustrated in Figure 9 and Figure 10. Most of the Zn_1−_*_x_*Mn*_x_*O nanostructure materials exhibit strong absorption in the UV and visible range and a strong UV emission [30].

### Density, specific surface area and crystallite size distribution

**Figure 11:**
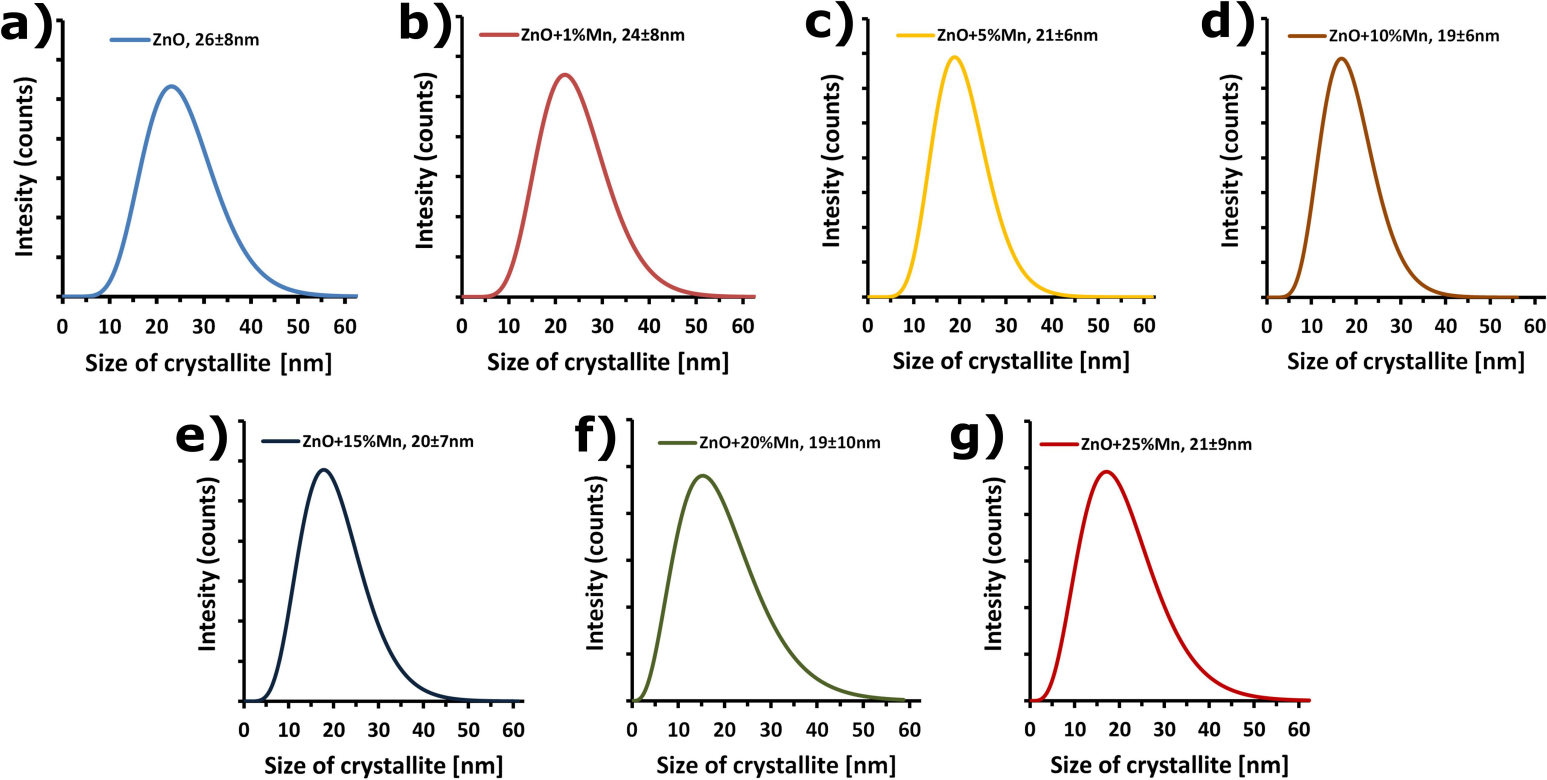
Crystallite size distribution obtained using Nanopowder XRD Processor Demo [68].

The theoretical density of ZnO is 5.606 g/cm^3^. The pycnometric density of the obtained reference sample of ZnO NPs without doping amounted to 5.09 g/cm^3^. The difference between the nano density and the theoretical density of ZnO can result above all from surface defects, the presence of Zn(OH)_2_ hydroxides and the non-stoichiometric composition. The density of Zn_1−_*_x_*Mn*_x_*O, irrespectively of the content of Mn^2+^ dopant, was constant at about 5.0 g/cm^3^. The lower density of Zn_1−_*_x_*Mn*_x_*O is related to the smaller atomic weight of Mn (54.93 u) in comparison with the Zn atoms (65.38 u) in Zn_1−_*_x_*Mn*_x_*O, and to the possible presence of a greater quantity of defects in the crystalline lattice. The specific surface area of Zn_1−_*_x_*Mn*_x_*O ranged from 40 to 70 m^2^/g (Table 5). The average size of Zn_1−_*_x_*Mn*_x_*O particles calculated based on the specific surface area ranged from 17 to 30nm (Table 5). Based on the XRD analysis using the Nanopowder XRD Processor Demo web application, the average size and the size distribution of crystallites were determined for Zn_1−_*_x_*Mn*_x_*O NPs (Figure 11). Zn_1−_*_x_*Mn*_x_*O material was characterised by an average crystallite size from 19 to 26 nm (Table 5), with a narrow distribution, which ranged from 30–35 nm to 40–60 nm depending on the dopant content (Figure 11). When comparing the obtained results from the Nanopowder XRD Processor Demo application and with that from Scherrer’s formula, similar crystallite sizes were obtained. The results fall within the range of the standard deviation of methods (Table 5). The calculations of the average crystallite size (XRD) coincide with the average particle sizes calculated based on the specific surface are with an accuracy of 2–6 nm, which confirms that:

- the nanoparticles are monocrystalline;
- the formed structures of nanoparticle conglomerates (Figure 2 and Figure 3) do not form aggregates;
- the size of crystallites is equal to the size of nanoparticles, thanks to which these expressions can be used interchangeably in this paper;
- the obtained crystallite size distribution may be interpreted as particle size distribution.

**Table 5:** Characteristic of the Zn_1−_*_x_*Mn*_x_*O NPs samples.

sample	specific surface area by gas adsorption, *a*_s_ ± σ [m^2^/g]	skeleton density by gas pycnometry, ρ_s_ ± σ [g/cm^3^]	average particle size from SSA BET, *d* ± σ [nm]	average crystallite size from Nanopowder XRD Processor Demo, *d* ± σ [nm]	average crystallite size from Scherrer’s formula	
					*d_a_* [nm]	*d_c_* [nm]

ZnO	42±1	5.09±0.04	28±1	26±8	22	28
Zn_0.99_Mn_0.01_O	40±1	4.98±0.05	30±1	24±8	21	26
Zn_0.95_Mn_0.05_O	50±1	4.95±0.03	24±1	21±6	18	19
Zn_0.90_Mn_0.10_O	60±1	5.03±0.04	20±1	19±6	17	15
Zn_0.85_Mn_0.15_O	57±1	5.01±0.04	21±1	20±7	18	15
Zn_0.80_Mn_0.20_O	70±1	4.97±0.03	17±1	19±10	16	12
Zn_0.75_Mn_0.25_O	63±1	4.97±0.04	19±1	21±9	19	13

## Conclusion

Zn_1−_*_x_*Mn*_x_*O (*x* = 0.01, 0.05, 0.10, 0.15, 0.2, 0.25) nanoparticles have been synthesized by microwave solvothermal synthesis. Zinc and manganese acetates dissolved in ethylene glycol were used as precursor. Crystalline Zn_1−_*_x_*Mn*_x_*O NPs with hexagonal wurtzite structure, pure in terms of phase, were obtained. Zn_1−_*_x_*Mn*_x_*O was characterised by an average particle size between 17 and 30 nm, a specific surface area between 40 and 70 m^2^/g and a density of circa 5.0 g/cm^3^. The average crystallite size was observed to decrease in line with an increase in the Mn^2+^ content. SEM images did not reveal an impact of the dopant on the spherical shape of nanoparticles. However, an impact of an increase in the Mn^2+^ dopant content on the shape of agglomerates and conglomerates of Zn_1−_*_x_*Mn*_x_*O nanoparticles was observed. The crystalline lattice parameters *a* and *c* in Zn_1−_*_x_*Mn*_x_*O increase in line with the dopant growth, which is a consequence of the greater ionic radius of Mn^2+^ dopant. The results confirm the usability of the developed method for obtaining crystalline Zn_1−_*_x_*Mn*_x_*O, pure in terms of phase, with a narrow particle size distribution with the nominal dopant content reaching 25 mol % and doping efficiency of circa 22%. Our paper also indicates a high potential of microwave solvothermal synthesis for obtaining doped ZnO nanoparticles.
